# Panel-Based Genetic Testing in a Consecutive Series of Individuals with Inherited Retinal Diseases in Australia: Identifying Predictors of a Diagnosis

**DOI:** 10.3390/genes16080888

**Published:** 2025-07-27

**Authors:** Alexis Ceecee Britten-Jones, Doron G. Hickey, Thomas L. Edwards, Lauren N. Ayton

**Affiliations:** 1 Department of Optometry and Vision Sciences, Faculty of Medicine, Dentistry and Health Sciences, University of Melbourne, Parkville 3010, Australia; layton@unimelb.edu.au; 2 Department of Surgery (Ophthalmology), Faculty of Medicine, Dentistry and Health Sciences, University of Melbourne, Parkville 3010, Australia; doron.hickey.1@unimelb.edu.au (D.G.H.); tom.edwards@unimelb.edu.au (T.L.E.); 3 Centre for Eye Research Australia, Royal Victorian Eye and Ear Hospital, Melbourne 3002, Australia

**Keywords:** genetic testing, targeted panels, next generation sequencing, retinal dystrophy, inherited retinal diseases, diagnostic utility, macular dystrophy, retinitis pigmentosa

## Abstract

**Background/Objectives**: Genetic testing is important for diagnosing inherited retinal diseases (IRDs), but further evidence is needed on the utility of singleton genetic testing in an Australian cohort. **Methods**: A consecutive series of individuals with clinically diagnosed IRDs without prior genetic testing underwent commercial panel-based sequencing (Invitae or Blueprint Genetics), clinical assessment, and multimodal imaging. Retinal images were graded using the Human Phenotype Ontology terms. Binary logistic regression was used to evaluate clinical predictors of a positive molecular diagnosis. **Results**: Among 140 participants (mean age 49 ± 19 years), genetic testing was undertaken, on average, 23 ± 17 years after the initial clinical IRD diagnosis. Of the 60% who received a probable molecular diagnosis, 40% require further phase testing, highlighting the limitations of singleton genetic testing. *USH2A*, *ABCA4*, and *RPGR* were the most common encountered genes; 67% of the probably solved participants had causative genes with targeted experimental treatments in ongoing human clinical trials. Symptom onset before the age of 30 (OR = 3.06 [95% CI: 1.34–7.18]) and a positive IRD family history (OR = 2.87 [95% CI: 1.27–6.78]) were each associated with higher odds of receiving a molecular diagnosis. Diagnostic rates were comparable across retinal imaging phenotypes (atrophy and autofluorescence patterns in widespread IRD, and the extent of dystrophy in macular IRDs). **Conclusions**: In an Australian IRD population without prior genetic testing, commercial panels yielded higher diagnostic rates in individuals with IRD onset before the age of 30 and those with an IRD family history. Further research is needed to understand the genetic basis of IRDs, especially isolated and late-onset cases, to improve diagnosis and access to emerging therapies.

## 1. Introduction

Inherited retinal diseases (IRDs) comprise a broad group of genetically heterogeneous disorders affecting around 1 in 2000–4000 people and, collectively, are the most common cause of vision impairment in working-age adults in many developed countries, including Australia and the United Kingdom [1,2]. The most common form of IRD is rod–cone dystrophy (also referred to as retinitis pigmentosa); other phenotypes include cone/cone–rod dystrophy, Leber congenital amaurosis, macular dystrophies, among others [3]. While visual impairment is the sole symptom in most IRD cases, approximately 20–30% of IRDs present alongside syndromic features, such as hearing loss, renal disease, metabolic disorders, or cardiac anomalies [4]. With a wide range of phenotypic features and overlap in clinical manifestations across different causative genes, genetic testing is essential for establishing an accurate IRD diagnosis.

To date, over 300 genes have been associated with IRDs [5]. In 2017, voretigene neparvovec-rzyl (LUXTURNA^®^), for treating biallelic *RPE65*-associated IRDs, became the first regulatory-approved gene therapy for any eye disease. As more gene therapies emerge [6], it is essential for people with IRDs to obtain a molecular diagnosis to determine their eligibility for upcoming genetic treatments. Having a molecular diagnosis also assists with targeted genetic counselling and family planning and empowers patients to seek options regarding management [7]. The emergence of potential gene therapy treatments has led to increased availability and implementation of next-generation sequencing approaches in clinical settings [8]. The most common first-tier testing approach for IRDs is panel-based testing, which can achieve a diagnostic yield of around 60% [9]. This yield varies depending on the IRD phenotype, sequencing technique, test settings, and ancestry [9].

Even though genetic testing for IRDs is recommended as the standard of care [10,11], in Australia, genetic testing for most IRDs is not covered by Medicare, Australia’s public healthcare system. Since 2021, the availability of industry-sponsored genetic testing programmes has improved access to genetic testing in both clinical ophthalmology and research settings [12,13,14,15,16,17]. While a number of studies have reported findings from panel-based genetic testing in IRDs in other countries [9,13,16,18,19,20,21], there is a lack of clear understanding regarding the clinical and phenotypic features that may be associated with higher diagnostic yields. Studies from the United Kingdom and Portugal have found that in non-syndromic RP, compared to genetically solved patients, unsolved patients more frequently had an age of disease onset above 30 years and were more likely to have atypical fundus features [22,23]. However, it remains unclear whether these findings extend to other populations, especially in Australia, where population-based next-generation sequencing testing has not been evaluated [9,24]. A clearer understanding of the utility of singleton testing in this context could assist clinicians in setting appropriate expectations and providing patient counselling.

This study aimed to evaluate the utility of singleton panel-based genetic testing and determine predictors of a molecular diagnosis in a consecutive series of participants with clinically suspected IRDs from an Australian research centre without prior genetic testing.

## 2. Materials and Methods

### 2.1. Ethics and Participants

Ethics approval was obtained from the Royal Victorian Eye and Ear Hospital Human Research and Ethics Committee (19/1443H) and the University of Melbourne Human Research Ethics Committee (#21037). The study was performed in accordance with the principles of the Declaration of Helsinki. All participants provided written informed consent to participate.

Participants were recruited as a part of the Victorian Evolution of inherited retinal diseases NaTUral history REgistry (VENTURE), a research study based at the University of Melbourne and the Centre for Eye Research Australia [25]. Participants comprised consecutive individuals enrolled in VENTURE until August 2023 who met the following criteria: (i) clinically diagnosed IRD phenotype with the potential for multiple causative genes, with clinical diagnosis confirmed by an ophthalmologist; (ii) no prior genetic testing; and (iii) consented to panel-based genetic testing. We excluded participants with IRD presentations specific to the characteristic of a single causative gene (e.g., choroideremia, X-linked retinoschisis) and female carriers of X-linked IRDs to avoid inflating estimated diagnostic yield and because alternative testing strategies may be more suitable.

### 2.2. Genetic Testing

Genetic testing was performed using next generation sequencing, employing either the Blueprint Genetics Retinal Dystrophy targeted gene panel (Blueprint Genetics, Finland; 322–351 nuclear and mitochondrial genes) or the Invitae Inherited Retinal Disorders Panel (Labcorp Genetics Inc., Burlington, NC, USA; 293–330 nuclear genes), with eligible participants processed through sponsored testing programmes. Both commercial laboratories are Clinical Laboratory Improvement Amendments (CLIA)-accredited, employing standardised processes for clinical molecular testing. Appendix A shows the list of IRD genes included in each targeted panel.

Saliva samples were collected according to laboratory-specific requirements. Both laboratories used a hybridization-based target capture method to target the regions of interest, which includes the coding exons and up to 20 base pairs outside of the exon–intron boundary of the flanking intronic sequence. Each laboratory had proprietary methods for detecting copy number variations, including single exon or larger deletions or duplications, and selected noncoding variants. Additionally, the Blueprint IRD panel captured up to 129 noncoding variants in 56 IRD genes and the Invitae IRD panel captured up to 3 noncoding variants in 2 genes. Both panels included additional sequencing of the highly repetitive, purine-rich sequence in the open-reading frame of exon 15 of the *RPGR* gene.

Bioinformatic analyses and variant curation were performed by each commercial laboratory according to their standard methods for clinical testing [26,27]. The genetic data presented incorporated any reclassifications issued up to 31 December 2023. Only pathogenic, likely pathogenic, and variants of uncertain significance were reported. Where required, reported variants were reassessed by a clinician with variant curation expertise (A.C.B.-J.). The genetic reports and clinical diagnoses were reviewed by a multidisciplinary team, which included ophthalmologists, optometrists with IRD expertise, genetic counsellors, and geneticists, to verify that the candidate gene matched the participants’ phenotype and reported inheritance pattern and that no additional potentially pathogenic variants were found in other common, likely candidate genes for their phenotype.

The participants’ initial IRD clinical diagnosis was documented based on the referral information and clinical history provided by their ophthalmologist. After genetic testing, the IRD diagnoses were re-assessed by the clinical team. The final diagnosis assignment was made based on a comprehensive evaluation of (i) genetic data, including identification of pathogenic or likely pathogenic variants in known IRD-associated genes; (ii) clinical assessment, including symptoms, age of onset, family history, ophthalmic examination findings, and retinal imaging; and (iii) genotype–phenotype correlation, matching the molecular findings with the observed clinical features and inheritance.

Participants were considered to have a probable molecular diagnosis if they had (1) one heterozygous pathogenic or likely pathogenic variants in a gene associated with a dominant IRD; (2) one hemizygous pathogenic or likely pathogenic variant in a gene associated with an X-linked IRD; or (3) two pathogenic or likely pathogenic variants (suspected or shown to lie on separate alleles) in a gene associated with a recessive IRD. For compound heterozygous variants with unknown phase, we used inferred phasing in gnomAD (v2.1.1), and a probable molecular diagnosis was only reported if the two variants have not been reported to occur on the same haplotype. Otherwise, participants were considered to have an inconclusive molecular diagnosis.

For this study, we defined “diagnostic yield” as the percentage of individuals who received a probable molecular diagnosis. Genetic counselling was provided to all participants, and those who required further testing were referred to clinical genetic services at a multidisciplinary ocular genetics centre.

### 2.3. Clinical and Phenotype Characteristics

Prospective clinical data was collected using a previously described protocol [25]. If a participant was unable to attend a clinical examination, retrospective retinal imaging and clinical records were obtained. Retinal images included ultra-widefield fundus imaging (Optos plc, Dunfermline, Scotland, United Kingdom) and spectral domain optical coherence tomography (OCT; Heidelberg Engineering, Heidelberg, Germany), and fundus autofluorescence (FAF) imaging captured using both instruments. Clinical information included best corrected visual acuity and Goldmann kinetic visual fields, and electroretinography, where available.

From available ultra-widefield and fundus autofluorescence retinal images, fundus features were graded based on retinal features, described using the ocular Human Phenotype Ontology terms (https://hpo.jax.org/ (accessed on 1 August 2024)) [28], to assess if diagnostic rates varied among subgroups exhibiting different retinal characteristics. We stratified cases into widespread retinal dystrophy (where the peripheral retina was affected equally or more than the central macula on ultra-widefield imaging) and macular dystrophies (where the central macular region was more severely affected than the periphery). We selected common retinal characteristics observed in each IRD group, consulting the existing literature [29,30,31,32,33]. Table 1 shows the retinal features, derived from the Human Phenotype Ontology terms, used to classify different IRD phenotypes in this study, along with our additional descriptions used to aid in the classification of retinal images. Fundus features were then independently graded by a clinician experienced in reviewing IRD images (A.C.B.-J.) and a medical retina ophthalmologist (D.H.), and classifications were determined by discussion.

In addition to common phenotype features, atypical phenotype features were noted using definitions by Birtel et al. [22]: significant interocular asymmetry in retinal phenotype; absence of bone spicule pigmentation in rod–cone dystrophies despite OCT showing thinning of the outer nuclear layer with ellipsoid zone loss; restriction of retinal atrophy to the far periphery; and atypical fundus autofluorescence pattern.

**Table 1:** Retinal features considered for phenotypic classification of widespread retinal dystrophy and macular dystrophy groups, based on the Human Phenotype Ontology terms.

Human Phenotype Ontology (HPO) Term Identifier	Human Phenotype Ontology (HPO) Term Name	Additional Descriptive Features Used in the Classification of Retinal Images in This Study
Widespread Retinal Dystrophy ^a^		
HP:0007737	Bone spicule pigmentation of the retina	Presence of distinct bone spicule pigmentation in any degrees of confluence (shown in Figure 1A,B)
HP:0007401	Macular atrophy	Presence of macular atrophy, reflecting loss of the retinal pigment epithelium with associated retinal photoreceptor loss, in the central macular isolated from any mid-peripheral atrophy (shown in Figure 1E,F)
HP:0200070	Peripheral retinal atrophy	Presence of discrete islands of sharply demarcated peripheral chorioretinal atrophy, interspersed by adjacent normal retina (shown in Figure 1G,H)
HP:0030602	Abnormal fundus autofluorescence imaging	Hyperfluorescent ring at the posterior pole, graded as: (i) regular perifoveal ring of hyperautofluorescence, seen as closed rings with an ellipsoid/round shape and regular borders (Figure 1I); (ii) mottled, irregular, or abnormal central macular autofluorescence without a distinct, regular ring (Figure 1J); (iii) no visible hyperautofluorescence in the macular or peri-macular region; (iv) Indistinguishable hyperautofluorescence pattern due to extensive atrophy (Figure 1K)
Macular Dystrophy ^a^		
HP:0030500	Yellow/white lesions of the macula	(i) Scattered macular lesions, including flecks, fundus flavimaculatus dystrophy, and speckled, reticular, and patterned hyperautofluorescence lesions, extending past the retinal arcades (shown in Figure 1L); (ii) Focal/isolated macular atrophy or lesions limited to the macula (shown in Figure 1M)

Note: ^a^ We identified widespread retinal dystrophies as cases where the peripheral retina was affected equally or more than the central macula on retinal images, and macular dystrophies as cases where the central macular region more severely affected than the periphery on retinal images. Figure 1 shows representative images of these phenotype characteristics referenced in the table.

### 2.4. Statistical Analysis

Statistical analysis was performed using R for statistical consulting (v. 4.2.2; R Core Team 2022). Differences in proportions between groups were compared using the two-tailed Fisher’s exact test.

Univariate and multivariate binary logistic regression was performed to investigate predictors of a molecular diagnosis from panel-based testing based on demographic and clinical variables. Predictor variables in the univariate logistic regression model included the following: age at symptom onset (under 30 versus 30 and over), sex, family history, laboratory (Blueprint/Invitae), time between symptom onset and clinical diagnosis (years), time between clinical and genetic diagnosis (years), hearing impairment, history of systemic conditions that could cause IRD-phenocopies (recorded as any history of cancer, retinotoxic medications, or autoimmune disease), atypical retinal phenotype, and level of visual impairment (stratified as mild [≤0.48 logMAR in better eye]; moderate [0.48–1.0 logMAR]; and severe [>1.0 logMAR]). Variables with *p* < 0.05 in univariate analyses were included in the multivariate model. Odds ratios with 95% confidence intervals were calculated. Model fit was assessed using the likelihood ratio test.

## 3. Results

### 3.1. Study Population

Between 1 May 2021 and 31 August 2023, 140 consecutive participants (from 124 unrelated families) with IRDs, without prior genetic testing, underwent commercial targeted-panel next generation sequencing, including 65 using the Blueprint Genetics retinal dystrophy panel and 75 using the Invitae inherited retinal disorders panel. Figure S1 details the included participants and reasons for exclusions among all participants enrolled in the VENTURE study during this period.

Participant demographics are shown in Table 2. Before genetic testing, most participants (68%) reported an initial clinical diagnosis of non-syndromic retinitis pigmentosa/rod–cone dystrophy, and 53% had no known family history of IRD.

Participants’ mean age at the time of genetic testing was 49 ± 19 years. On average, participants experienced a time gap of 23 ± 17 years between receiving a clinical IRD diagnosis and undergoing genetic testing.

### 3.2. Diagnostic Outcomes

Of 140 cases, a probable molecular diagnosis was identified in 84 participants (71/124 unrelated families), representing a diagnostic yield of 60%. Figure 2 shows the causative genes among participants who received a probable molecular diagnosis, and the distribution of phenotypes associated with each causative gene, alongside the types of pathogenic variants found within each genotype–phenotype group. Table S1 shows the known candidate variants identified.

Among these participants, 40% (34/84) required additional testing to confirm the phase of compound heterozygous variants. Inheritance patterns were predominantly autosomal recessive (58%), with autosomal dominant and X-linked accounting for 21% and 20% of solved cases, respectively. The most common causative genes were *USH2A* (18%), *ABCA4* (n = 15%), and *RPGR* (n = 15%), together accounting for 48% of the probably solved cases (Figure 2A).

A total of 94 unique known pathogenic and likely pathogenic causative variants were reported across 25 genes associated with IRDs (Figure 2B). Missense variants were most common, accounting for 42% of unique causative variants, followed by frameshift (28%) and nonsense (13%) variants. Copy number variations (8%), splice (4%), and intronic variants (4%) made up 16% of the unique variants.

### 3.3. Diagnostic Utility

In addition to confirming their IRD clinical diagnosis, the genetic findings offered additional clinical utility for 70% (59 out of 84) of the cases that received a probable diagnosis. Thirteen cases initially diagnosed as rod–cone dystrophy or achromatopsia were reclassified as follows: seven to macular or cone–rod dystrophy, two to congenital stationary night blindness, two to Jalili syndrome, and one to each Usher and Refsum syndromes [35]. Two thirds (67%) of the 84 participants who received a genetic diagnosis had causative genes with targeted experimental treatments registered in human clinical trials (*ABCA4*, *GUCY2D*, *NR2E3*, *PDE6B*, *PRPF31*, *RHO*, *RPGR*, *USH2A*).

Of the 56 unsolved cases, 48% were carriers of one pathogenic variant in an autosomal recessive gene matching phenotype, and 13% had a highly suspicious VUS requiring further evidence to determine pathogenicity, either heterozygous in presumed autosomal dominant cases or homozygous in presumed autosomal recessive cases. One of the unsolved cases had a novel homozygous variant of significance in *RPE65* (c.260A > G, p.Asp87Gly), which was not present in population databases and has not been reported in the literature. No other candidate variants were found in this individual. The individual’s phenotype raised high suspicion of an *RPE65*-related IRD (Figure S2). They have been referred for further research studies to investigate the pathogenicity of the VUS to determine if they could be a candidate for voretigene neparvovec-rzyl gene therapy.

### 3.4. Diagnostic Yield Based on Clinical Diagnosis and Imaging Features

Figure 3 shows the diagnostic yield by clinical and demographic features. Higher diagnostic rates were observed in individuals with likely X-linked inheritance (compared to autosomal inheritance), a family history of IRD (compared to simplex cases), and self-reported European ethnicity (from Australia, New Zealand, and Europe, compared to other regions).

**Figure 1:**
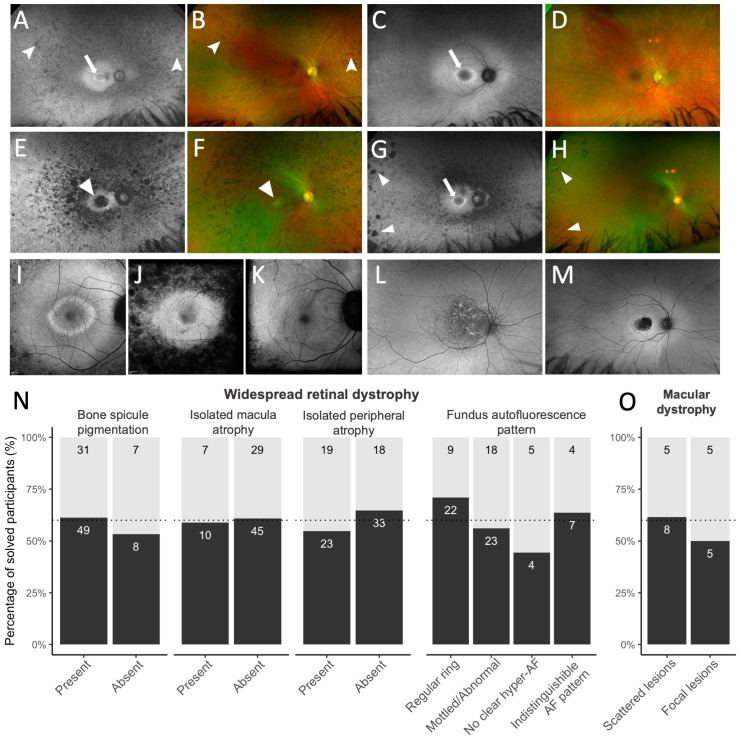
Representative images of retinal phenotype features for widespread (**A**–**K**) and macular dystrophies (**L**,**M**), classified based on the Human Phenotype Ontology terms described in Table 1, and diagnostic rates among these different retinal phenotype features (**N**). (**A**,**B**) Bone spicule pigmentation (HP:0007737; arrowheads) and a regular parafoveal autofluorescence ring (HP:0030629, white arrow), in an *USH2A*-related IRD. (**C**,**D**) Absence of bone spicule pigmentation with a regular parafoveal autofluorescence ring (HP:0030629, white arrow), in an *USH2A*-related IRD. (**E**,**F**) Isolated central atrophy (HP:0007401; arrowhead) in an *RP1*-related IRD. (**G**,**H**) Peripheral chorioretinal atrophy (HP:0200070; triangles) and a regular parafoveal autofluorescence ring (HP:0030629, white arrow) in an unsolved case. (**I**–**K**) Representative images of abnormal fundus autofluorescence classifications (HP:0030602) captured using 488 nm autofluorescence imaging, shown as (**I**) a regular parafoveal autofluorescent ring, (**J**) mottled abnormal hyperfluorescence without a distinct ring pattern, and (**K**) no visible hyperautofluorescence ring. (**L**,**M**) Macular dystrophy were classified as having either (**L**) scattered hyperautofluorescence lesions extending beyond the retinal arcades or (**M**) focal/isolated macula lesion within the retinal arcades. (**N**) Proportion of participants with widespread retinal dystrophy who received a genetic diagnosis (features shown in panels (**A**–**K**)). (**O**) Proportion of participants with macular dystrophy who received a genetic diagnosis (features shown in panels (**L**,**M**)). Ungradable images were excluded.

**Figure 2:**
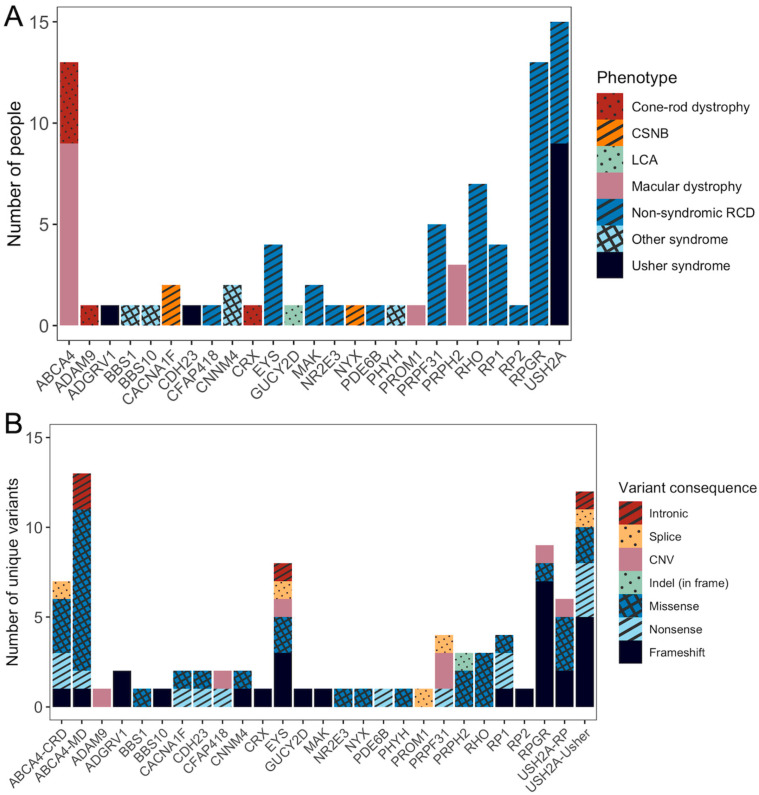
Molecular diagnosis of 84 participants who received a probable diagnosis through singleton targeted panel genetic testing. (**A**) Number of individuals identified with variants in each causative genes and their associated inherited retinal disease phenotypes. (**B**) Number and type of unique variants identified for each causative gene, stratified by genetic and phenotype groups.

**Figure 3:**
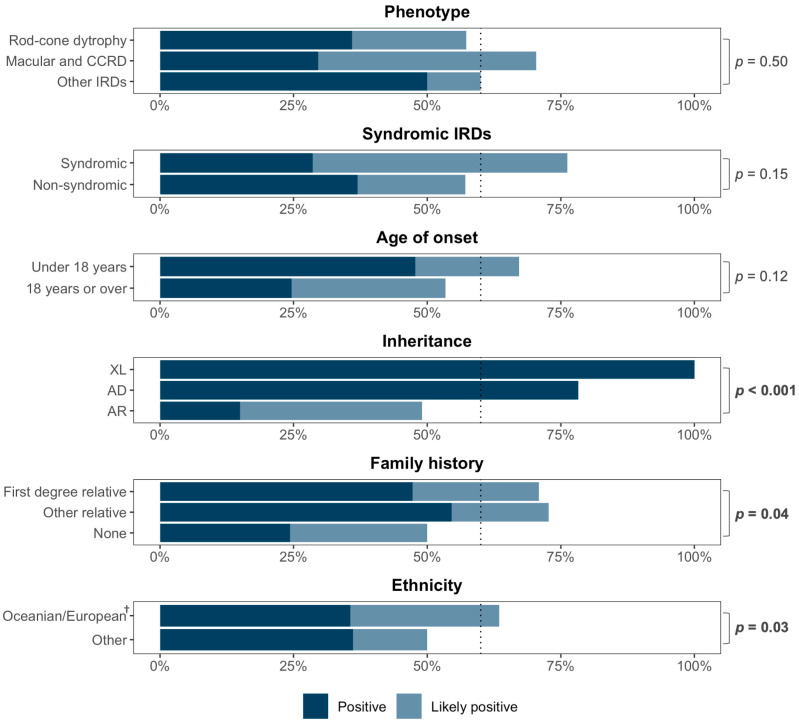
Diagnostic yield of targeted panel sequencing according to clinical and demographic characteristics. *p*-values were calculated using Fisher’s exact test, comparing positive/likely positive outcomes against no molecular diagnosis. ^†^ Oceanian/European backgrounds included participants who self-reported their ethnicity as being of European descent, including those from Australia, New Zealand, Northwest Europe, Southern Europe, and Eastern Europe. Abbreviations: AD = autosomal dominant. AR = autosomal recessive. CCRD = cone/cone–rod dystrophy. IRD = inherited retinal disease. XL = X-linked.

Figure 1N shows the diagnostic yield stratified by common phenotypic features graded from retinal images, separated for widespread dystrophies and macular dystrophies. Among participants with widespread retinal dystrophy (n = 98), the diagnostic rate was similar between all the phenotype features graded (bone spicule pigmentation, macular chorioretinal atrophy, peripheral chorioretinal atrophy). Compared to cases with regular hyperautofluorescent rings (71%), fewer cases with abnormal/mottled hyperautofluorescence (56%) or no hyperautofluorescent rings (44%) received a molecular diagnosis, but the proportions were not significantly different (*p* = 0.12). In macular dystrophies, diagnostic rates were similar between cases with hyperautofluorescence lesions extending beyond the retinal arcades (62%) and cases with only isolated macular lesions or atrophy within the arcades (50%).

Atypical fundus features were noted in 11 of 140 participants [22], including substantial asymmetry of the fundus changes (n = 1), restriction of retinal atrophy to the far periphery (n = 1), atypical AF patterns (n = 4), or absence of bone spicule pigmentation in rod–cone dystrophy despite OCT showing thinning of the outer nuclear layer with loss of ellipsoid zone (n = 5).

### 3.5. Predictors of Diagnosis

Univariate and multivariate binary logistic regression were used to evaluate predictors of a molecular diagnosis from clinical and phenotype features (Table 3).

Both analyses showed that individuals with age of symptom onset before 30 years (OR = 3.06 [95% CI: 1.34–7.18]) and a positive IRD family history (OR = 2.87 [95% CI: 1.27–6.78]) were more likely to receive a probable molecular diagnosis from singleton panel-based testing, and individuals with atypical phenotypes were less likely to obtain a diagnosis (OR = 0.26 [95% CI: 0.07–0.85]). European ethnicity was associated with higher odds of a molecular diagnosis in the univariate analysis compared to non-European ethnicity (OR = 2.57 [95% CI: 1.16–5.79]), but the association was not significant in the multivariate analysis.

## 4. Discussion

This study shows the benefits and limitations of singleton panel-based testing in a consecutive cohort of IRD participants in Australia without prior genetic testing. Commercial panel-based testing achieved a diagnostic yield of 60%, comparable to previously reported international cohorts [9,18,21,36,37]. However, when considering only those who received a definitive diagnosis without requiring further testing, the diagnostic yield based on existing known variants is 36%. Individuals with symptom onset prior to age 30 and those with a positive family history each had three times higher odds of obtaining a molecular diagnosis from targeted IRD panels as a first-tier test. Among participants receiving a putative molecular diagnosis, 13% had their IRD reclassified, for some leading to modified management of extraocular features in syndromic IRDs, and 67% had causative genes with targeted experimental treatments registered in human clinical trials.

Almost half (40%) the participants with a putative molecular diagnosis require further testing to resolve the phase of recessive variants, highlighting the limitations of singleton testing in IRDs. This consideration is important for clinicians when setting patient expectations pre-test and to assess the need for family testing to obtain a conclusive genetic diagnosis. Engaging in family testing has other potential benefits. Trio testing could facilitate the detection of de novo variants and allow phasing of compound heterozygous variants during interpretation, rather than post-test [38]. Segregation testing to evaluate the inheritance pattern of novel variants could generate further evidence supporting their pathogenicity. However, family samples may not always be available, especially if testing occurs later in life. Additional research comparing the benefits and risks of singleton versus trio testing in this population, considering cost, turnaround time, and potential implications on family dynamics, could further guide their applications in clinical settings.

The predisposition of a positive result in IRDs with a family history and younger onset age had been reported by several other studies [18,23,24,39]. It has been suggested that cases with an onset of over 50 years of age could have a multigenic or multifactorial aetiology [39]. Younger individuals may also be more likely to harbour severe variants, such as nonsense or truncating variants that lead to loss of function and earlier symptom onset. With increased access to genetic testing among younger people, variants with earlier manifestations may have already been reported [40]. Additional investigations are needed to understand the pathogenicity of late-onset IRDs, including the involvement of other epigenetic risk factors. We followed definitions by Birtel et al. to identify cases with atypical presentations [22]. Consistent with findings in their cohort from the United Kingdom, atypical phenotypes were associated with lower diagnostic rates. It remains unclear whether specific phenotypic features among unsolved cases potentially indicate undiscovered gene-phenotype associations or mechanisms. There is also a clinical overlap between IRDs and other retinal diseases, including autoimmune retinopathy and age-related macular degeneration, further complicating diagnosis [41,42]. To improve consistency in phenotyping, ontological foundations developed to provide robust terminology standards and enhance data interoperability [28]. As knowledge about genotype–phenotype correlations expands, developing consensus on the nomenclature of phenotypes and clinical features within disease classes could improve uniformity in utilising structured phenotype data to assist in the interpretation of genotype-disease classifications.

Compared to international cohorts, the three most encountered genes in this population, *USH2A*, *ABCA4*, and *RPGR*, mirror those observed in large population-based IRD cohorts from the United Kingdom [43,44], the United States of America [3,17], Germany [45], and Argentina [46], demonstrating similarities in common causative genes across these populations. We observed a higher diagnostic yield among participants reporting European ethnicity (65%) compared to those of non-European descent (42%) in our cohort. This finding aligns with recent studies from non-European populations, such as from Korea [47], China [48,49], and Japan [50], reporting lower diagnostic yields (40–55%) in mixed IRD cohorts using targeted panels or exome-based approaches. However, given the diverse range of non-European ethnicities represented in our cohort, further investigation into their genetic associations is needed to provide clearer insights into the molecular causes of IRDs across different ethnic groups. Our study also represents an older age group, as we included consecutive patients enrolled in a research registry who had not previously undergone genetic testing. This cohort likely represents individuals who would not have accessed genetic testing in clinical settings due to lack of public funding or being a non-priority population (e.g., not engaging in family planning).

Globally, there is significant variation in the degree to which different countries and healthcare systems can provide IRD genetic testing as part of clinical service [8,51,52]. Patients in the UK can access genetic testing through a National genomic testing initiative [51], and in Europe there are various national initiatives for genetic testing, as well as academic hospital laboratories and industry partners [53]. In the USA, implementation of sponsored genetic testing programmes, such as the ‘ID your IRD’ programme by Invitae, based on a partnership with Spark Therapeutics, and the My Retina Tracker Genetic testing programme by the Foundation Fighting Blindness [16], has significantly increased access to genetic testing, mainly by subsidising costs.

The availability of commercial panel-based testing approaches offers an efficient first-tier testing strategy [18,54] and is increasingly being used in countries without publicly funded ocular genetic testing, such as Australia. In a study from the USA, implementation of no-cost genetic testing programmes increased access by 29%, mainly by subsidising costs [12]. However, strategies are needed for further testing, including family testing and research on negative results. Clinicians should also be aware of the different coverage of IRD genes in different panels [15]. Notably, over 60% of the unsolved cases had at least one variant matching the phenotype that could benefit from further investigation, either to identify a second variant for autosomal recessive IRDs or to gather additional evidence to understand the pathogenicity of a high-suspicion VUS.

There are several considerations for interpreting the current study. We achieved similar diagnostic yields across two commercial laboratories; however, both had different regions of noncoding variant targeting. Singleton genetic testing was conducted due to limited resources, and we could not further test family members within this study to confirm the phase of autosomal recessive variants. However, phasing was inferred based on co-occurrence in population databases to strengthen the probability of the variant interpretation.

## 5. Conclusions

In an Australian IRD population without prior access to genetic testing, commercial targeted panel testing helped resolve genetic diagnoses and identify potential candidates for future targeted therapies, bridging a 23-year gap between clinical diagnosis and genetic testing in this cohort. However, approximately half of those diagnosed needed additional family or phase testing, highlighting a key consideration for setting outcome expectations.

Our findings also underscore the importance of collaboration between IRD research and clinical genetics services for comprehensive care. A significantly lower diagnostic yield was found in isolated cases without an IRD family history, with onset after the age of 30, and with atypical fundus features, highlighting gaps in our understanding of the genetic causes of IRDs and the need for further research to improve diagnosis and potential treatment access.

## Figures and Tables

**Table 2:** Participant demographic characteristics.

Characteristic (n = 140)	Summary
Age at genetic testing, years	49 ± 19
IQR	33–66
**Sex**	
Male	81 (58%)
Female	59 (42%)
**Age at symptom onset**, years	23 ± 19
IQR	8–36
**Mean disease duration**, years	27 ± 18
IQR	12–39
**Family history of IRD**	
Parent and/or sibling	53 (38%)
Other relative	13 (9%)
No known family history	74 (53%)
**Ethnicity** ^a^	
North African and Middle Eastern	10 (7%)
North-West European	11 (8%)
Oceanian	82 (59%)
Peoples of The Americas	1 (1%)
South-East Asian	7 (5%)
Southern and Central Asian	10 (7%)
Southern and Eastern European	11 (8%)
Sub-Saharan African	2 (1%)
Mixed ethnicities	6 (4%)
**Inherited retinal disease diagnosis**	
Rod–cone dystrophy/Retinitis pigmentosa	84 (60%)
Macular dystrophy	19 (14%)
Syndromic IRD	21 (15%)
Cone/cone–rod dystrophy	8 (6%)
Other IRD (achromatopsia, congenital stationary night blindness, Leber congenital amaurosis)	8 (6%)

Descriptive data are shown as n (%). Continuous data are shown as mean ± standard deviation, and interquartile range (IQR) is additionally provided. ^a^ Ethnicity was classified based on the Australian Standard Classification of Cultural and Ethnic Groups (2019) [34]. Abbreviations: IRD = inherited retinal disease.

**Table 3:** Univariate and multivariate binary logistic regression on factors associated with the likelihood of receiving a probable molecular diagnosis from commercial panel-based genetic testing (n = 140 participants).

	Univariate Analysis		Multivariate Analysis	
	OR (95% CI)	*p*-Value	OR (95% CI)	*p*-Value
**Age at symptom onset** (<30 years)	**2.57 (1.24, 5.41)**	0.012 *	**3.06 (1.34, 7.18)**	0.009 **
Laboratory (Invitae)	0.62 (0.31, 1.22)	0.17	-	
Sex (male)	1.19 (0.60, 2.35)	0.62	-	
**Ethnicity** (European)	**2.57 (1.16, 5.79)**	0.020 *	2.16 (0.86, 5.53)	0.10
**Positive family history**	**2.47 (1.24, 5.06)**	0.011 *	**2.87 (1.27, 6.78)**	0.013 *
Time between symptom onset and clinical diagnosis (years)	0.99 (0.94, 1.03)	0.54	-	
Time between clinical and genetic diagnosis (years)	1.00 (0.98, 1.02)	0.82	-	
Hearing impairment	0.89 (0.41, 1.95)	0.76	-	
Potential indicators for phenocopies ^a^	0.40 (0.15, 1.00)	0.051	-	
**Atypical phenotype** ^b^	**0.28 (0.08, 0.85)**	0.03 *	**0.26 (0.07, 0.85)**	0.031 *
Disease stage by visual acuity ^c^				
Early/mild	Ref		-	
Moderate	2.01 (0.82, 5.26)	0.14	-	
Advanced	1.29 (0.56, 3.03)	0.55	-	

Statistically significant predictors are in bold (* *p* ≤ 0.05; ** *p* ≤ 0.0). Abbreviations: OR, odds ratio. CI, confidence interval. ^a^ History of cancer, retinotoxic medications, and/or autoimmune disease. ^b^ Atypical presentations defined as unilaterality or substantial asymmetry of the fundus changes, restriction of retinal atrophy to the far periphery, atypical AF patterns, sharply demarcated mid-peripheral chorioretinal atrophy, or absence of bone spicule pigmentation in rod–cone dystrophy. ^c^ Disease stages by visual acuity: early/mild (≤0.48 [6/18 or 20/60] in better eye); moderate (between 0.48 and 1.0 LogMAR [6/60 or 20/200]), and severe (>1.0 [6/60 or 20/200])).

## Data Availability

Causative variants are provided in Table S1. Non-identifiable, aggregated data supporting the findings of this study are available from the corresponding author (A.C.B.-J.) upon reasonable request for ethically approved projects.

## Supplementary Materials

The following supporting information can be downloaded at: https://www.mdpi.com/article/10.3390/genes16080888/s1. Figure S1: Flow chart of participants from the Victorian Evolution of inherited retinal diseases NaTUral history REgistry (VENTURE) who were contacted to participate in the study. Figure S2: Retinal images of a case with autosomal recessive rod–cone dystrophy, which was classified as unsolved but is suspected to have *RPE65*-related inherited retinal diseases. Table S1: Cases with a probable causative variant identified.

## Abbreviations

The following abbreviations are used in this manuscript:

FAF	Fundus autofluorescence
HPO	Human Phenotype Ontology
IRD	Inherited retinal disease
OCT	Optical coherence tomography
RCD	Rod–cone dystrophy
VENTURE	Victorian Evolution of inherited retinal diseases NaTUral history REgistry
VUS	Variant of Uncertain Significance

## Appendix A

**Table A1:** Gene lists included in the commercial targeted panels.

Genes on Both Blueprint and Invitae Targeted Panels					
*ACO2*	*ADAM9*	*ADAMTS18*	*ADGRV1*	*ADIPOR1*	*AGBL5*
*AHI1*	*AIPL1*	*ALMS1*	*ARHGEF18*	*ARL13B*	*ARL2BP*
*ARL3*	*ARL6*	*ARMC9*	*ARSG*	*ATF6*	*ATOH7*
*B9D1*	*BBIP1*	*BBS1* ^†^	*BBS10*	*BBS12*	*BBS2*
*BBS4* ^†^	*BBS5* ^†^	*BBS7*	*BBS9*	*BEST1* ^†^	*C1QTNF5*
*C2ORF71/PCARE*	*C5ORF42/CPLANE1*	*C8ORF37/CFAP418*	*CA4*	*CABP4*	*CACNA1F*
*CACNA2D4*	*CAPN5*	*CC2D2A*	*CDH23*	*CDH3*	*CDHR1*
*CEP164*	*CEP19*	*CEP250*	*CEP290* ^†,§^	*CEP41*	*CEP78*
*CEP83* *	*CERKL*	*C21ORF2* ^†^*/CFAP410*	*CHM* ^†^	*CIB2*	*CISD2*
*CLN3* ^†^	*CLN5* *^,‡^	*CLN6* *^,†,‡^	*CLN8* *^,‡^	*CLRN1* ^†^	*CNGA1*
*CNGA3* ^†^	*CNGB1*	*CNGB3*	*CNNM4*	*COL11A1* ^†^	*COL11A2*
*COL18A1*	*COL2A1* ^†^	*COL9A1*	*COL9A2*	*COL9A3*	*CRB1*
*CRX*	*CSPP1*	*CTNNA1*	*CTSD* *^,‡^	*CWC27*	*CYP4V2*
*DHDDS* ^†^	*DHX38*	*DRAM2*	*DTHD1*	*EFEMP1*	*ELOVL4*
*EMC1*	*EXOSC2* *	*EYS* ^†^	*FAM161A*	*FLVCR1*	*FRMD7* ^†^
*FZD4*	*GNAT1*	*GNAT2* ^†^	*GNB3*	*GNPTG* ^†^	*GPR143* *^,†^
*GPR179*	*GRM6*	*GUCA1A*	*GUCY2D* ^†^	*HARS*	*HGSNAT* ^†^
*HK1* ^†^	*HMX1*	*IDH3A*	*IDH3B*	*IFT140* ^†^	*IFT172*
*IFT27*	*IFT81*	*IMPDH1*	*IMPG1*	*IMPG2*	*INPP5E*
*INVS*	*IQCB1*	*JAG1* ^†^	*KCNJ13*	*KCNV2*	*KIAA0586*
*KIAA1549*	*KIF11*	*KIF7*	*KIZ*	*KLHL7*	*LCA5*
*LRAT* ^†^	*LRIT3*	*LRP2*	*LRP5*	*LZTFL1*	*MAK*
*MERTK*	*MFN2*	*MFRP*	*MFSD8* ^‡^	*MKKS*	*MKS1*
*MTTP* ^†^	*MYO7A* ^†^	*NAGLU* *^,‡^	*NDP* ^†^	*NEK2*	*NMNAT1* ^†^
*NPHP1*	*NPHP3*	*NPHP4*	*NR2E3*	*NR2F1*	*NRL*
*NYX*	*OAT*	*OCA2* *^,†^	*OFD1* ^†^	*OPA1* ^†^	*OPA3*
*OPN1SW* *	*OTX2*	*P3H2*	*PAX2*	*PCDH15* ^†^	*PCYT1A*
*PDE6A*	*PDE6B*	*PDE6C* ^†^	*PDE6D*	*PDE6G*	*PDE6H*
*PDZD7*	*PEX1*	*PEX10*	*PEX11B*	*PEX12*	*PEX13*
*PEX14*	*PEX16*	*PEX19*	*PEX2*	*PEX26*	*PEX3*
*PEX5*	*PEX6* ^†^	*PEX7* ^†^	*PHYH*	*PITPNM3*	*PLA2G5*
*PLK4* *	*PNPLA6*	*POC1B*	*POMGNT1*	*PPT1* *^,†,‡,§^	*PRCD*
*PRDM13* ^†^	*PROM1* ^†^	*PRPF3*	*PRPF31* ^†^	*PRPF4*	*PRPF6*
*PRPF8*	*PRPH2*	*PRPS1*	*RAB28*	*RAX2*	*RBP3*
*RBP4*	*RCBTB1*	*RD3*	*RDH11*	*RDH12*	*RDH5* ^†^
*REEP6*	*RGR*	*RGS9*	*RGS9BP*	*RHO*	*RIMS1*
*RLBP1*	*ROM1*	*RP1*	*RP1L1*	*RP2*	*RPE65* ^†^
*RPGR* (including *ORF15*) ^†,‡^	*RPGRIP1* ^†^	*RPGRIP1L*	*RS1*	*RTN4IP1*	*SAG*
*SAMD11*	*SCLT1*	*SDCCAG8*	*SEMA4A*	*SGSH* *^,†,‡^	*SLC24A1*
*SLC45A2* *	*SLC7A14*	*SNRNP200*	*SPATA7*	*SPP2*	*TCTN1*
*TCTN2*	*TCTN3*	*TEAD1*	*TIMM8A* ^†^	*TIMP3*	*TMEM107*
*TMEM126A*	*TMEM138*	*TMEM216*	*TMEM231* ^†^	*TMEM237*	*TMEM67*
*TOPORS*	*TPP1* *^,†^	*TRAF3IP1*	*TREX1*	*TRIM32*	*TRPM1*
*TSPAN12*	*TTC21B*	*TTC8*	*TTLL5*	*TTPA*	*TUB*
*TUBGCP4* *	*TUBGCP6* *	*TULP1*	*TYR* *^,†^	*TYRP1* *	*USH1C*
*USH1G*	*USH2A* ^†^	*VCAN*	*VPS13B*	*WDPCP*	*WDR19*
*WFS1* ^†^	*ZNF408*	*ZNF423*	*ZNF513*		
Genes Only on Blueprint Retinal Dystrophy Panel					
*B9D2*	*CEP104*	*CEP120*	*CPE*	*CTC1*	*CTNNB1*
*DFNB31*	*ESPN*	*FDXR*	*GRK1*	*KIAA0556*	*KIAA0753*
*MMACHC*	*MT-ATP6*	*MT-ATP8*	*MT-CO1*	*MT-CO2*	*MT-CO3*
*MT-CYB*	*MT-ND1*	*MT-ND2*	*MT-ND3*	*MT-ND4*	*MT-ND4L*
*MT-ND5*	*MT-ND6*	*MT-RNR1*	*MT-RNR2*	*MT-TA*	*MT-TC*
*MT-TD*	*MT-TE*	*MT-TF*	*MT-TG*	*MT-TH*	*MT-TI*
*MT-TK*	*MT-TL1*	*MT-TL2*	*MT-TM*	*MT-TN*	*MT-TP*
*MT-TQ*	*MT-TR*	*MT-TS1*	*MT-TS2*	*MT-TT*	*MT-TV*
*MT-TW*	*MT-TY*	*MVK* ^†^	*PANK2* ^†^	*PISD*	*SCAPER*
*SLC25A46*	*SRD5A3*	*TUBB4B*	*YME1L1*	*ABCD1* *	*AMACR* *
*ARR3* *	*COQ2* *	*DNAJC5* *	*DYNC2H1* *^,†^	*ISPD* *	*LAMA1* *
*PDSS1* *	*PDSS2* *				
Genes Only on Invitae Inherited Retinal Disorders Panel					
*ACBD5*	*ADGRA3*	*AHR*	*ASRGL1*	*CCT2*	*CLCC1*
*CLUAP1*	*FSCN2*	*GDF6*	*GUCA1B*	*IFT43*	*IFT80*
*MAPKAPK3*	*MPDZ*	*NEUROD1*	*PAX6*	*TRNT1*	*UNC119*
*WHRN*	*ADAMTSL4* ^‡^	*C10orf11* ^‡^*/LRMDA*	*C12orf65* ^‡^*/MTRFR*	*DHX32* ^‡^	*DNAJC17* ^‡^
*DSCAML1* ^‡^	*ERCC6 ^‡^*	*FBLN5* ^‡^	*GNS* ^‡^	*GPR45* ^‡^	*GRN* ^‡^
*HMCN1* ^‡^	*IFT74* ^‡^	*IFT88* ^‡^	*ITM2B* ^‡^	*LYST* ^‡^	*MIR204* ^‡^
*MTPAP* ^‡^	*NBAS* ^‡^	*OR2W3* ^‡^	*POC5* ^‡^	*RBP1* ^‡^	*RP9* ^‡^
*SIX6* ^‡^	*SLC24A5* ^‡^	*TMED7* ^‡^	*WDR34* ^‡^		

* Included in Blueprint 351 gene panel and not previous versions. † Selected non-coding variants covered in Blueprint panel. ‡ Included in Invitae 330 gene panel and not previous versions. § Selected non-coding variants covered by Invitae panel.
