# Supplementary material for: Panel-Based Genetic Testing in a Consecutive Series of Individuals with Inherited Retinal Diseases in Australia: Identifying Predictors of a Diagnosis
Source: Genes (Basel). 2025 Jul 27;16(8):888. doi: 10.3390/genes16080888 (PMC12385882; doi:10.3390/genes16080888)

Britten-Jones AC, Hickey DG, Edwards TL, Ayton LN. Panel-based genetic testing in a consecutive series of individuals with inherited retinal diseases in Australia: Identifying predictors of a diagnosis.

Table S1: Cases with a probable causative variant identified.

Case ID	Clinical diagnosis	Gene	Transcript	Variant 1				Variant 2				Variant co-occurrence
				Variant	Consequence	Zygosity	ACMG class	Variant	Consequence	Zygosity	ACMG class	
1	MD	*ABCA4*	NM_000350.2	c.1609C>T (p.Arg537Cys)	Missense	Hom	P	c.5881G>A (p.Gly1961Arg)	Missense	Hom	P	Not reported
2	MD	*ABCA4*	NM_000350.2	c.161G>A (p.Cys54Tyr)	Missense	Het	P	c.2588G>C (p.Gly863Ala)[†]	Missense	Het	P-low penetrance[†]	No
3	MD	*ABCA4*	NM_000350.2	c.1995C>A (p.Tyr665*)	Nonsense	Het	P	c.5882G>A (p.Gly1961Glu)	Missense	Het	P-low penetrance	No
4	MD	*ABCA4*	NM_000350.2	c.3056C>T (p.Thr1019Met)	Missense	Het	P	c.6079C>T (p.Leu2027Phe)	Missense	Het	P	No
5	MD	*ABCA4*	NM_000350.2	c.3056C>T (p.Thr1019Met)	Missense	Het	P	c.5196+1137G>A (Intronic)	Intronic	Het	P	Not reported
6	MD	*ABCA4*	NM_000350.2	c.4222T>C (p.Trp1408Arg)	Missense	Het	P	c.4205dup (p.Leu1403Phefs*19)	Frameshift	Het	P	No
7	CRD	*ABCA4*	NM_000350.2	c.4222T>C (p.Trp1408Arg)	Missense	Het	P	c.2939T>A (p.Leu980*)	Nonsense	Het	P	Not reported
8	MD	*ABCA4*	NM_000350.2	c.428C>T (p.Pro143Leu)	Missense	Het	P	c.4383G>C (p.Trp1461Cys)	Missense	Het	P	No
9	MD	*ABCA4*	NM_000350.2	c.428C>T (p.Pro143Leu)	Missense	Het	P	c.4383G>C (p.Trp1461Cys)	Missense	Het	P	No
10	MD	*ABCA4*	NM_000350.2	c.5461-10T>C (Intronic)	Intronic	Het	P	c.2588G>C (p.Gly863Ala)[‡]	Missense	Het	P-low penetrance[‡]	Not reported
11[§]	CRD	*ABCA4*	NM_000350.2	c.5461-10T>C (Intronic)	Intronic	Het	P	c.716G>A (p.Trp239*)	Nonsense	Het	P	Not reported
12[§]	CRD	*ABCA4*	NM_000350.2	c.6181_6184del (p.Thr2061Serfs*53)	Frameshift	Het	P	c.4577C>T (p.Thr1526Met)	Missense	Het	P	Not reported
13	CRD	*ABCA4*	NM_000350.2	c.6816+1G>A	Splice	Het	P	c.5435T>A (p.Ile1812Asn)	Missense	Het	LP	No
14	CRD	*ADAM9*	NM_003816.3	c.(410+1_411-1)_(*1_?)del	CNV	Hom	LP					
15	Usher	*ADGRV1*	NM_032119.3	c.2849del (p.Gly950Glufs*30)	Frameshift	Het	P	c.6245_6249del (p.Thr2082Serfs*18)	Frameshift	Het	P	Not reported
16[§]	BBS	*BBS1*	NM_024649.4	c.1169T>G (p.Met390Arg)	Missense	Hom	P					
17	BBS	*BBS10*	NM_024685.4	c.271dup (p.Cys91Leufs*5)	Frameshift	Hom	P					
18	CSNB	*CACNA1F*	NM_005183.4	c.2905C>T (p.Arg969*)	Nonsense	Hemi	P					
19	CSNB	*CACNA1F*	NM_005183.4	c.3052G>A (p.Gly1018Arg)	Missense	Hemi	P					
20	Usher	*CDH23*	NM_022124.5	c.5237G>A (p.Arg1746Gln)	Missense	Het	P	c.7908C>G (p.Tyr2636*)	Nonsense	Het	P	Not reported
21	RCD	*CFAP418*	NM_177965.4	c.26T>A (p.Leu9*)	Nonsense	Het	LP	g.96252231_96259922delins[96255731_96255865inv]	CNV	Het	LP	Not reported
22	Jalili	*CNNM4*	NM_020184.4	c.707G>A (p.Arg236Gln)	Missense	Hom	LP					
23	Jalili	*CNNM4*	NM_020184.3	c.86del (p.Leu29Argfs*21)	Frameshift	Hom	P					
24[§]	CRD	*CRX*	NM_000554.6	c.429del (p.Pro145Leufs*42)	Frameshift	Het	P					
25	RCD	*EYS*	NM_001142800.2	c.6192-1G>A	Splice	Het	P	c.910dup (p.Trp304Leufs*9)	Frameshift	Het	P	Not reported

26	RCD	*EYS*	NM_001142800.2	c.6714del (p.Ile2239Serfs*17)	Frameshift	Het	P	c.5836-3C>T	Intronic	Het	P	No
27§	RCD	*EYS*	NM_001142800.2	c.7228G>T (p.Ala2410Ser)	Missense	Het	P	c.8054G>A (p.Gly2685Glu)	Missense	Het	LP	No
28	RCD	*EYS*	NM_001142800.2	c.8408dup (p.Asn2803Lysfs*9)	Frameshift	Het	P	c.(2259+1_2260-1)_(2846+1_2847-1) del	CNV	Het	P	Not reported
29	LCA	*GUCY2D*	NM_000180.4	c.389del (p.Pro130Leufs*36)	Frameshift	Het	P	c.2302C>T (p.Arg768Trp)	Missense	Het	P	No
30	RCD	*MAK*	NM_001242957.3	c.1297_1298insAlu (p.Lys433insAlu)	Frameshift	Hom	P					
31	RCD	*MAK*	NM_001242957.3	c.1297_1298insAlu (p.Lys433insAlu)	Frameshift	Hom	P					
32	RCD	*NR2E3*	NM_014249.3	c.767C>A (p.Ala256Glu)	Missense	Hom	P					
33	CSNB	*NYX*	NM_022567.2	c.551T>C (p.Leu184Pro)	Missense	Hemi	LP					
34	RCD	*PDE6B*	NM_000283.3	c.2401C>T (p.Gln801*)	Nonsense	Hom	P					
35¶	Refsum	*PHYH*	NM_006214.3	c.823C>T (p.Arg275Trp)	Missense	Hom	P					
36	MD	*PROM1*	NM_006017.3	c.1632G>T (p.Gly544=)	Synonymous	Hom	P					
37	RCD	*PRPF31*	NM_015629.3	c.(?_-1)_(*1_?)del	CNV	Het	P					
38§	RCD	*PRPF31*	NM_015629.4	c.(?_-396)_(*1_?)del	CNV	Het	P					
39	RCD	*PRPF31*	NM_015629.3	c.1147-2A>G (Splice acceptor)	Splice	Het	P					
40§	RCD	*PRPF31*	NM_015629.3	c.841C>T (p.Gln281*)	Nonsense	Het	P					
41	RCD	*PRPF31*	NM_015629.3	c.841C>T (p.Gln281*)	Nonsense	Het	P					
42	MD	*PRPH2*	NM_000322.4	c.461_463del (p.Lys154del)	Indel (in frame)	Het	P					
43	MD	*PRPH2*	NM_000322.4	c.634A>C (p.Ser212Arg)	Missense	Het	P					
44	MD	*PRPH2*	NM_000322.5	c.646C>T (p.Pro216Ser)	Missense	Het	P					
45§	RCD	*RHO*	NM_000539.3	c.328T>C (p.Cys110Arg)	Missense	Het	P					
46	RCD	*RHO*	NM_000539.3	c.328T>C (p.Cys110Arg)	Missense	Het	P					
47§	RCD	*RHO*	NM_000539.3	c.328T>C (p.Cys110Arg)	Missense	Het	P					
48§	RCD	*RHO*	NM_000539.3	c.44A>G (p.Asn15Ser)	Missense	Het	P					
49§	RCD	*RHO*	NM_000539.3	c.44A>G (p.Asn15Ser)	Missense	Het	P					
50	RCD	*RHO*	NM_000539.3	c.568G>T (p.Asp190Tyr)	Missense	Het	P					
51	RCD	*RHO*	NM_000539.3	c.568G>T (p.Asp190Tyr)	Missense	Het	P					
52	RCD	*RP1*	NM_006269.1	c.1012C>T (p.Arg338*)	Nonsense	Hom	P					
53	RCD	*RP1*	NM_006269.1	c.1012C>T (p.Arg338*)	Nonsense	Hom	P					
54	RCD	*RP1*	NM_006269.1	c.2172_2185del (p.Ile725Argfs*6)	Frameshift	Het	P					
55	RCD	*RP1*	NM_006269.1	c.539T>G (p.Phe180Cys)	Missense	Het	P	c.742C>T (p.Gln248*)	Nonsense	Het	P	Not reported

56	RCD	*RP2*	NM_006915.3	c.383_386del (p.Phe128Cysfs*27)	Frameshift	Hemi	LP					
57	RCD	*RPGR*	NM_001034853.2	c.823G>C (p.Gly275Arg)	Missense	Hemi	P					
58	RCD	*RPGR*	NM_001034853.2	c.823G>C (p.Gly275Arg)	Missense	Hemi	P					
59	RCD	*RPGR*	NM_001034853.1	complex structural rearrangement exons 13-15	CNV	Hemi	P					
60	RCD	*RPGR*	NM_001034853.1	complex structural rearrangement exons 13-15	CNV	Hemi	P					
61	RCD	*RPGR*	NM_001034853.2	c.2236_2237del (p.Glu746Argfs*23)	Frameshift	Hemi	P					
62	RCD	*RPGR*	NM_001034853.2	c.2252_2255del (p.Lys751Argfs*63)	Frameshift	Hemi	P					
63	RCD	*RPGR*	NM_001034853.2	c.2252_2255del (p.Lys751Argfs*63)	Frameshift	Hemi	P					
64	RCD	*RPGR*	NM_001034853.2	c.2252_2255del (p.Lys751Argfs*63)	Frameshift	Hemi	P					
65	RCD	*RPGR*	NM_001034853.2	c.2405_2406del (p.Glu802Glyfs*32)	Frameshift	Hemi	P					
66	RCD	*RPGR*	NM_001034853.2	c.2442_2445del (p.Gly817Lysfs*2)	Frameshift	Hemi	P					
67	RCD	*RPGR*	NM_001034853.2	c.2635del (p.Glu879Lysfs*210)	Frameshift	Hemi	P					
68	RCD	*RPGR*	NM_001034853.2	c.3039_3040del (p.Glu1014Glyfs*64)	Frameshift	Hemi	P					
69	RCD	*RPGR*	NM_001034853.2	c.3364dup (p.Met1122Asnfs*22)	Frameshift	Hemi	P					
70	Usher	*USH2A*	NM_206933.2	c.10073G>A (p.Cys3358Tyr)	Missense	Het	P	c.10626del (p.Glu3542Aspfs*8)	Frameshift	Het	P	Not reported
71	Usher	*USH2A*	NM_206933.2	c.11411del (p.Pro3804Leufs*13)	Frameshift	Het	P	c.12067-2A>G (Splice acceptor)	Splice	Het	P	Not reported
72	Usher	*USH2A*	NM_206933.2	c.1606T>C (p.Cys536Arg)	Missense	Het	P	Deletion (Exon 27)	CNV	Het	P	Not reported
73	RCD	*USH2A*	NM_206933.2	c.2276G>T (p.Cys759Phe)	Missense	Het	P	c.14166del (p.Ser4723Valfs*23)	Frameshift	Het	P	Not reported
74	RCD	*USH2A*	NM_206933.3	c.2276G>T (p.Cys759Phe)	Missense	Het	P	c.12525G>T (p.Trp4175Cys)	Missense	Het	P	Not reported
75§	RCD	*USH2A*	NM_206933.3	c.2276G>T (p.Cys759Phe)	Missense	Het	P	c.6163G>A	Missense	Het	LP	No
76§	RCD	*USH2A*	NM_206933.3	c.2276G>T (p.Cys759Phe)	Missense	Het	P	c.6163G>A	Missense	Het	LP	No
77	RCD	*USH2A*	NM_206933.2	c.2276G>T (p.Cys759Phe)	Missense	Het	P	Gain (Exons 62-63)	CNV		LP	Not reported
78	RCD	*USH2A*	NM_206933.2	c.2276G>T (p.Cys759Phe)	Missense	Het	P	c.1679del (p.Pro560Leufs*31)	Frameshift	Het	P	No
79	Usher	*USH2A*	NM_206933.2	c.2299del (p.Glu767Serfs*21)	Frameshift	Hom	P					
80	Usher	*USH2A*	NM_206933.2	c.2299del (p.Glu767Serfs*21)	Frameshift	Het	P	c.8981G>A (p.Trp2994*)	Nonsense	Het	P	No
81	Usher	*USH2A*	NM_206933.2	c.7595-2144A>G (Intronic)	Intronic	Het	P	c.9676C>T (p.Arg3226*)	Nonsense	Het	P	Not reported

82	Usher	*USH2A*	NM_206933.2	c.920_923dup (p.His308Glnfs*16)	Frameshift	Het	P	c.11103T>A (p.Tyr3701*)	Nonsense	Het	P	Not reported
83	Usher	*USH2A*	NM_206933.2	c.920_923dup (p.His308Glnfs*16)	Frameshift	Het	P	c.11103T>A (p.Tyr3701*)	Nonsense	Het	P	Not reported
84	Usher	*USH2A*	NM_206933.2	c.920_923dup (p.His308Glnfs*16)	Frameshift	Het	P	c.11336del (p.Tyr3779Leufs*5)	Frameshift	Het	P	Not reported

Variant co-occurrence (inferred phasing) information obtained from in gnomAD v2.1.1. Not reported indicates that at least one of the variants was not present in gnomAD.
Abbreviations: BBS=Bardet-Biedl Syndrome. CRD=Cone-rod dystrophy. CSNB=Congenital stationary night blindness. Hemi=Hemizygous. Het=Heterozygous. Hom=Homozygous. Jalili=Jalili syndrome. LCA=Leber congenital amaurosis. MD=Macular dystrophy. RCD=rod-cone dystrophy. Refsum=Refsum disease. Usher=Usher syndrome.

† The research team subsequently identified a heterozygous *ABCA4* c.5603A>T (p.Asn1868Ile) variant in this case, which was not reported in the clinical report. As phase remains unresolved, this case is classified as probably solved.

‡ The research team subsequently identified a homozygous *ABCA4* c.5603A>T (p.Asn1868Ile) variant in this case, which was not reported in the clinical report.

§ Previously reported in Britten-Jones et al (2024) Ophthalmol Sci; DOI: 10.1016/j.xops.2024.100649.

¶ Previously reported in Truong et al (2024) Clin Exp Optom; DOI: 10.1080/08164622.2024.2401509.

Figure S1. Flow chart of participants from the Victorian Evolution of inherited retinal diseases NaTUral history REgistry (VENTURE) who were contacted to participate in the study

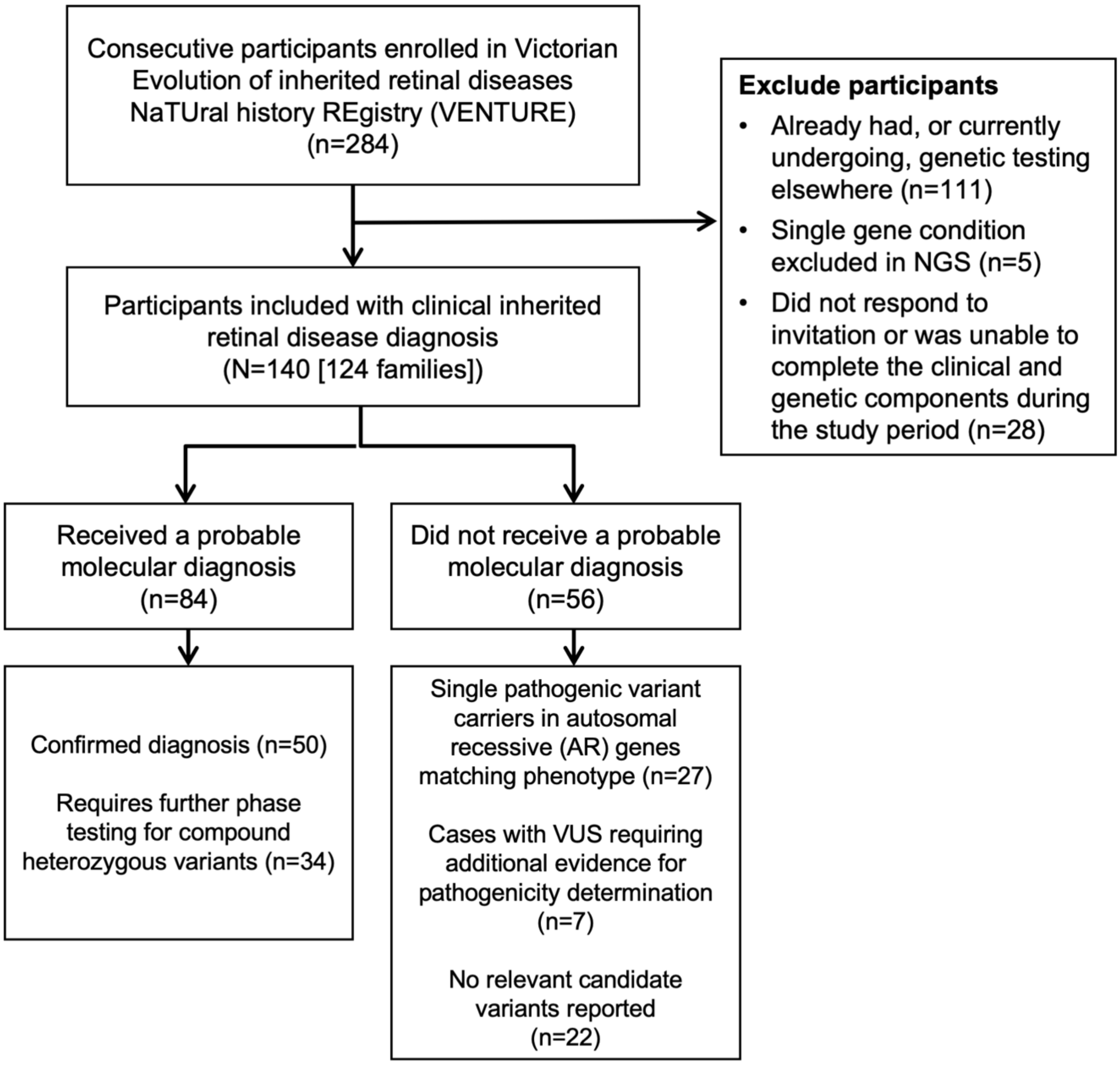

Figure S2. Retinal images of a case with autosomal recessive rod-cone dystrophy, which was classified as unsolved, but is suspected to have *RPE65*-related inherited retinal diseases. A novel homozygous variant in *RPE65* c.260A>G (p.Asp87Gly) was identified, which was not present in population databases and has not been reported in the literature in individuals with *RPE65*-related conditions (NM_000329.2). No other candidate variants were found in this individual. The *RPE65* variant is predicted to be pathogenic using *in silico* tools (Revel=0.91. SIFT=0 [deleterious]. PolyPHEN=0.947 [probably damaging]). This male participant was diagnosed with rod-cone dystrophy (retinitis pigmentosa) at 16 years old, and had low myopia and visual acuities of 20/80 [6/24] and 20/120 [6/36] at the time of testing (24 years old). Full field and multifocal electroretinograms showed no discernible responses to scotopic or photopic stimuli. (A) Retinal imaging on fundus colour images showing bone spicule pigmentation and a rod-cone dystrophy phenotype. (B) Fundus autofluorescence images show severely diminished fundus autofluorescence signals, suggesting a possible diagnosis of *RPE65*-related inherited retinal disease. (C) optical coherence tomography showing ellipsoid zone loss. The participant has been referred for further functional research studies to investigate the pathogenicity of the VUS.

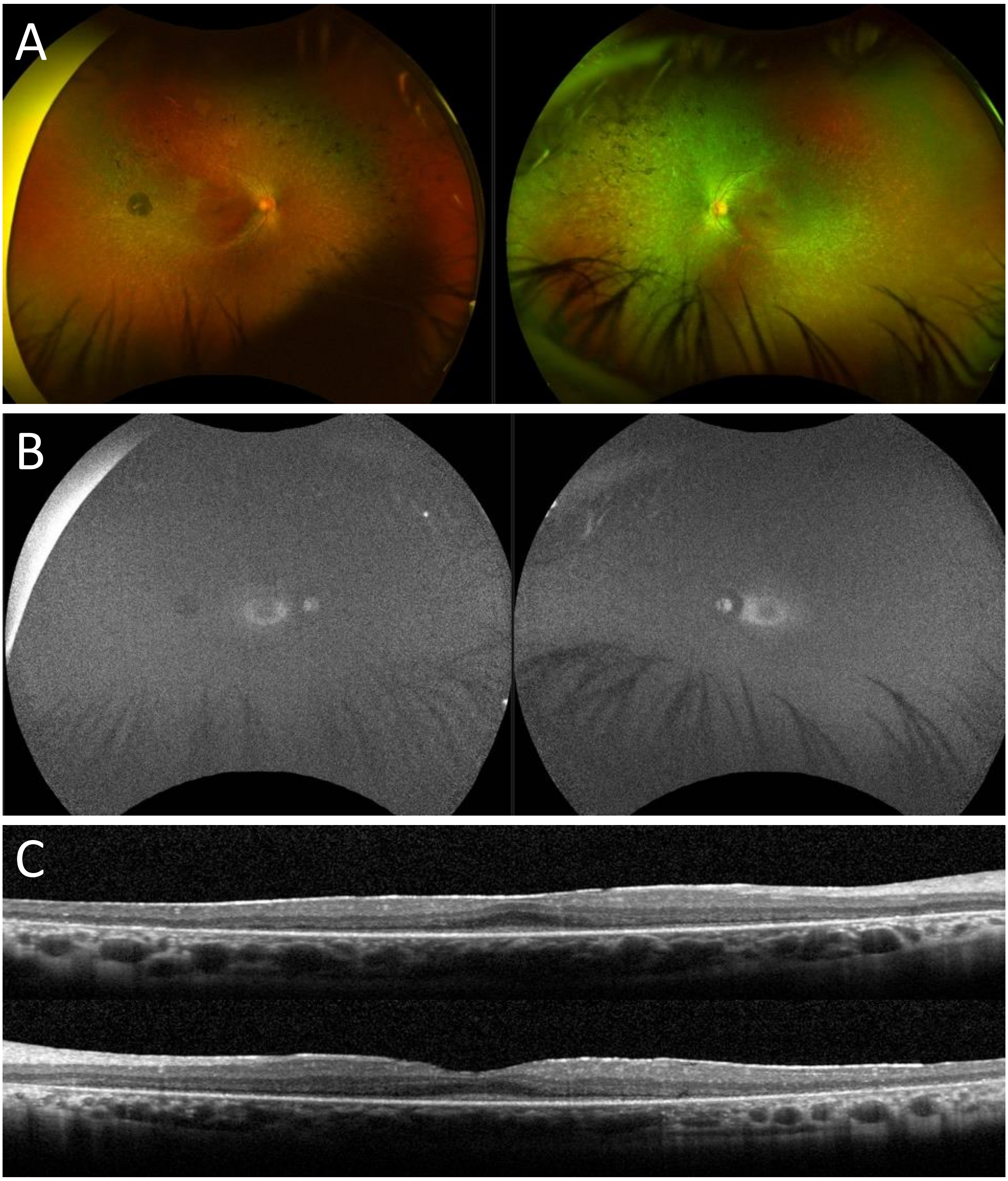

Supplement: Supplementary file 1 [file genes-16-00888-s001.zip › genes-3753886-Supplemental materials.pdf]
